# Phosphine and carbene azido-cations: [(L)N_3_]^+^ and [(L)_2_N_3_]^+^†
†Electronic supplementary information (ESI) available: Details on VT NMR experiments, preparation of **5**, quantum chemical calculations and X-ray crystallography. CCDC 1403531–1403535. For ESI and crystallographic data in CIF or other electronic format see DOI: 10.1039/c5sc02336j


**DOI:** 10.1039/c5sc02336j

**Published:** 2015-07-21

**Authors:** Daniel Winkelhaus, Michael H. Holthausen, Roman Dobrovetsky, Douglas W. Stephan

**Affiliations:** a Department of Chemistry , University of Toronto , 80 St. George St , Toronto , Ontario M5S3H6 , Canada . Email: dstephan@chem.utoronto.ca; b School of Chemistry , Raymond and Beverly Sackler Faculty of Exact Sciences , Tel Aviv University , Tel Aviv 69978 , Israel

## Abstract

The cationic N_3_-species [(*p*-HC_6_F_4_)_3_PN_3_]^+^ (**1**) featuring a perfluoro-arene phosphonium group serves as a N_3_^+^-source in stoichiometric reactions with several Lewis bases (L) allowing for the stepwise formation of [(L)N_3_]^+^ and [(L)_2_N_3_]^+^ cations (L = phosphine, carbene) with liberation of (*p*-HC_6_F_4_)_3_P. X-Ray diffraction analysis and computational studies provide insight into the bonding in these remarkably stable azido-cations.

## Abstract 2

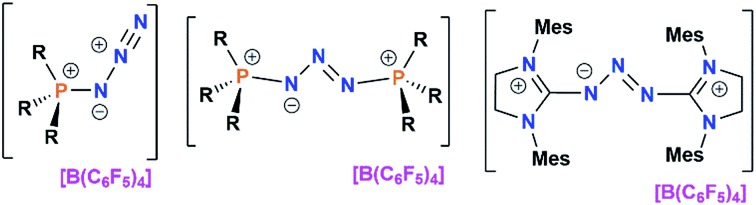
The cationic N_3_-species [(*p*-HC_6_F_4_)_3_PN_3_]^+^ (**1**) featuring a perfluoro-arene phosphonium group serves as a N_3_^+^-source in stoichiometric reactions with several Lewis bases (L) allowing for the stepwise formation of [(L)N_3_]^+^ and [(L)_2_N_3_]^+^ cations (L = phosphine, carbene) with liberation of (*p*-HC_6_F_4_)_3_P.

## Introduction

A major component of the recent renaissance in p-block chemistry^1^ has been the use of neutral, two electron donors like carbenes or phosphines for the stabilization of homoatomic, low valent main group element fragments.^2^ Most recently Cummins used an anthracene stabilized P_2_ for the synthesis of an aromatic [P_2_N_3_].^3^ Species of the form (L)_2_E_2_ (E = B,^4^ C,^5^ Si,^6^ Ge,^7^ Sn,^8^ N,^9^ P,^10^ As,^11^ Sb;^12^ L = carbene, phosphine) have been prepared affording ligand stabilization of unique diatomic main group fragments. In addition, the groups of Robinson, Bertrand and Roesky have exploited these donors for isolation of dications and radical cations of type [(carbene)_2_E_2_]^*n*+^ (E = C,^5^ P,^13^ As;^14^*n* = 1, 2) while Burford, Weigand, Jones and Grützmacher prepared [(carbene)_2_P_3_]^+^^15^ or bicyclic [(L)_2_P_4_]^2+^ species (L = carbene, AsPh_3_, PPh_3_).^16^ The latter compound is of special interest since L can be exchanged for more basic donors. A similar donor exchange was reported for [(Ph_3_P)PPh_2_]^+^ affording [(carbene)PPh_2_]^+^.^17^ The nature of these and related systems, especially the L–E and E–E bonding, has sparked vigorous debate.^18^

Our recent studies of highly electrophilic phosphonium cations (EPCs) have demonstrated that species such as the fluorophosphonium cation [(C_6_F_5_)_3_PF]^+^^19^ exhibit remarkable Lewis acidity and thus act as effective catalysts in a range of Lewis acid mediated transformations.^20^ At the same time, we were motivated to probe the utility of these EPCs as synthons for cationic azides. Herein we describe the synthesis of [(*p*-HC_6_F_4_)_3_PN_3_]^+^ (**1**) and its use as a synthon to species of the form [(L)N_3_]^+^ and [(L)_2_N_3_]^+^ (L = phosphine, carbene).

## Results and discussion

**Scheme 1:**
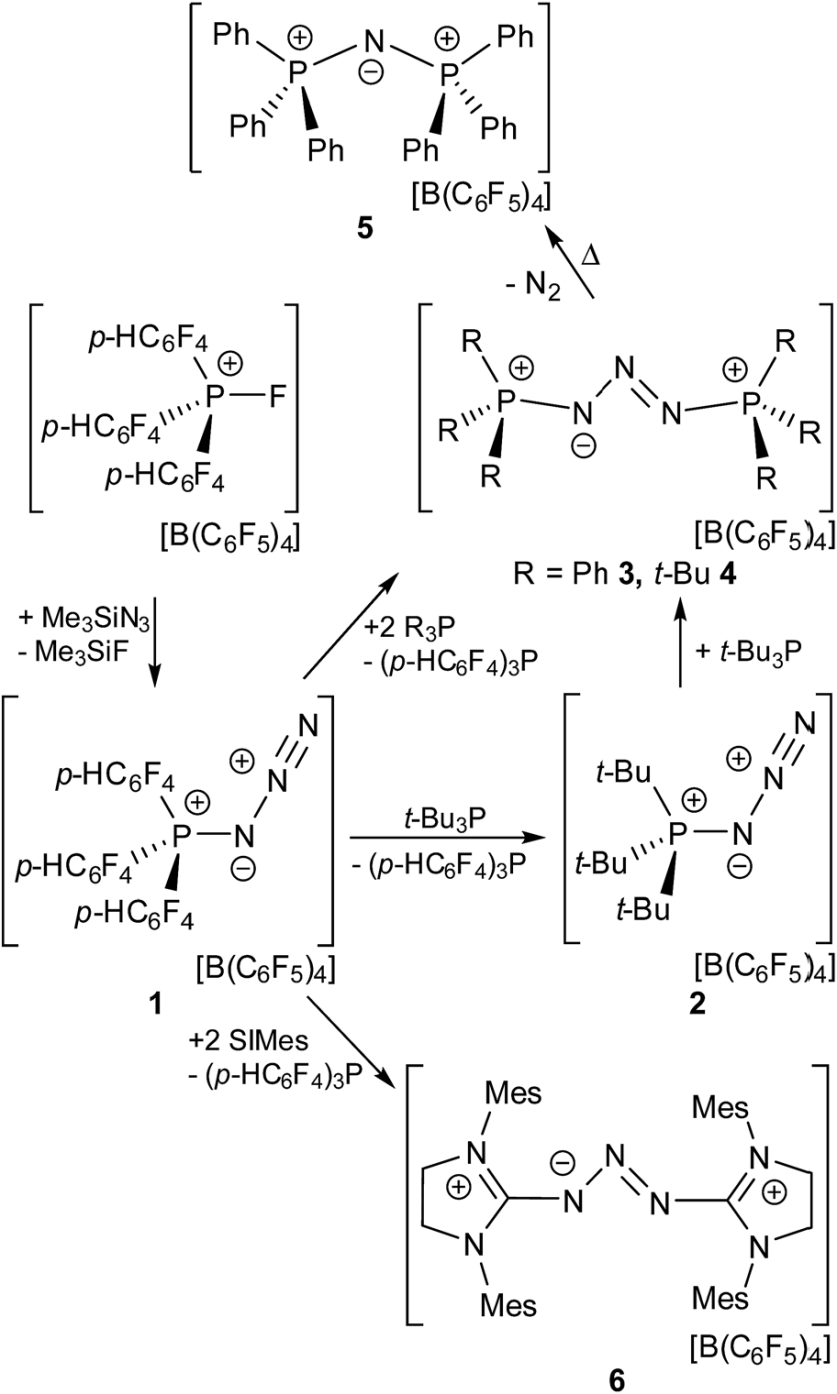
Preparation of **1–6**.

Compound [(*p*-HC_6_F_4_)_3_PN_3_][B(C_6_F_5_)_4_] (**1**) was readily prepared in quantitative yield by the reaction of [(*p*-HC_6_F_4_)_3_PF][B(C_6_F_5_)_4_] with Me_3_SiN_3_ in CH_2_Cl_2_ (Scheme 1).§
§Extra precaution must be taken when working with azides in CH_2_Cl_2_ solution as they are potentially dangerous and can result in the possible formation of dangerous and explosive diazidomethane. The ^31^P{^1^H} NMR spectrum of **1** in CD_2_Cl_2_ gives rise to a broad singlet resonance (*δ*(^31^P) = 17.5 ppm, Δ*ν*_1/2_ = 30 Hz).

**Fig. 1:**
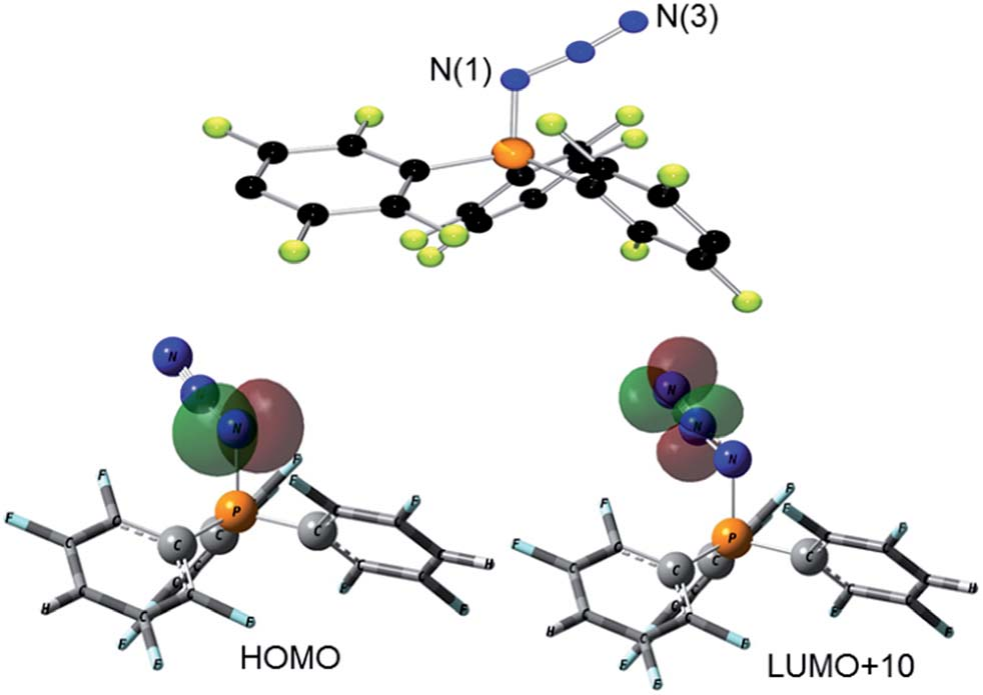
POV-ray depiction of the cation in **1** (top). Hydrogen atoms are omitted for clarity. Selected bond distances and angles: P(1)–N(1) 1.651(2), P(1)–C(13) 1.783(2), P(1)–C(1) 1.789(2), P(1)–C(7) 1.789(2), N(1)–N(2) 1.260(2), N(2)–N(3) 1.114(2), N(1)–P(1)–C(13) 111.6(2), N(1)–P(1)–C(1) 102.3(2), C(13)–P(1)–C(1) 112.6(2), N(1)–P(1)–C(7) 109.8(2), C(13)–P(1)–C(7) 110.5(2), C(1)–P(1)–C(7) 109.7(2), N(2)–N(1)–P(1) 120.9(2), N(3)–N(2)–N(1) 172.4(2). Graphical presentation of the HOMO and LUMO+10 of **1** calculated at the wB97XD/def2-TZV level of theory (surface iso-value = 0.05).

The presence of the *para* hydrogen substituents in **1** is crucial as the fully fluorinated derivative [(C_6_F_5_)_3_PF]^+^ is prone to nucleophilic attack at this position.^21^ The formulation of compound **1** was subsequently confirmed by an X-ray crystallographic study (Fig. 1).

The molecular structure of **1** shows a distorted tetrahedral environment about the phosphorus atom with angles at P ranging from 102.3(1)–112.6(1)°. The P(1)–N(1) bond length of 1.651(2) Å is shorter than those observed in azido-substituted phosphenium cations (1.67 Å)^22^ and azido-phosphines (1.73 Å),^23^ consistent with the electron deficient nature of the phosphonium center and the strongly polarized nature of the P–N bond. The azido moiety shows an N(1)–N(2)–N(3) angle that slightly deviates from the ideal 180° (172.4(2)°) while the N(1)–N(2) (1.260(2) Å) and N(2)–N(3) bond length (1.114(2) Å) are in the expected range for azide substituents. Compound **1** is a rare example of a crystallographically characterized salt of a cationic azido-phosphonium species^24^ while several neutral, azido-substituted P(iii) and P(v) derivatives have been reported to date.^25^ Other examples of monocationic nitrogen species, reported in the literature, include diazonium ions,^26^ the homoleptic [N_5_]^+^ of Christe,^27^ [N_2_F]^+^,^28^ Hünig's stable triazenium ions [R_2_N_3_R_2_]^+^^29^ and aminodiazonium ions of the form [(R_2_N)N_2_]^+^^30^ (R = H, silyl, alkyl, aryl).

To gain further insights into the nature of **1**, the geometry of the cation was optimized at the wB97XD/def2-TZV level of theory (see ESI†). The *p*-type HOMO of **1** is located at the P-bonded N atom while the LUMO is part of two sets of degenerated π*-type orbitals involving the *p*-HC_6_F_4_-groups (LUMO to LUMO+9). The first accessible acceptor orbital is a π*-type orbital located at the terminal N_2_ moiety (LUMO+10). This stands in contrast to other EPCs where the accessible acceptor orbital involves the P–F σ* orbital at the P center.^19^ The corresponding P–N σ* orbital in **1** is much higher in energy (LUMO+11). NBO analysis reveals that the P–N single bond in **1** is occupied by 1.92 electrons, while the P-bound N atom features two lone pairs of p and sp^0.69^ type. Donation of electron density from these lone pairs into σ* orbitals of the adjacent P–C bonds occurs (LP(p)_N1_ → σ*P–C7 11.2 kcal mol^–1^, LP(p)_N1_ → σ*P–C7 7.4 kcal mol^–1^, LP(sp^0.69^)_N1_ → σ*P–C7 3.7 kcal mol^–1^). The secondary interactions of these lone pairs may account for the observed shortening of the P–N bond in **1** in comparison to other P-azido species.^22^,^23^

Compound **1** reacts with the Lewis base *t*-Bu_3_P in CH_2_Cl_2_ solution (Scheme 1).§ The ^31^P{^1^H} NMR spectrum of the reaction mixture showed a septet resonance at *δ*(^31^P) = –72.3 ppm (^3^*J*_PF_ = 36.4 Hz)19*b* which corresponds to (*p*-HC_6_F_4_)_3_P and a new singlet resonance at low field (*δ*(^31^P) = –85.9 ppm) which is in the typical chemical shift range for an *N*-substituted trialkyl-phosphonium derivative.^31^ Collectively, these observations indicate the formal transfer of the N_3_^+^-moiety in **1** to *t*-Bu_3_P yielding [*t*-Bu_3_PN_3_][B(C_6_F_5_)_4_] (**2**) which was isolated in 98% yield. The nature of **2** was confirmed by X-ray single crystallography and the metrics were found similar to **1** (see ESI†). Interestingly, **2** and **1** are very stable and do not show any signs of degradation on heating in C_6_D_5_Br solution to 100 °C for 24 h. Attempts to independently synthesize **2** from the reaction of [*t*-Bu_3_PF][B(C_6_F_5_)_4_]^19^ with Me_3_SiN_3_ failed even after prolonged reaction times at 100 °C in C_6_D_5_Br solution. This reflects the increased steric demand of the [*t*-Bu_3_PF]^+^ cation.

**Fig. 2:**
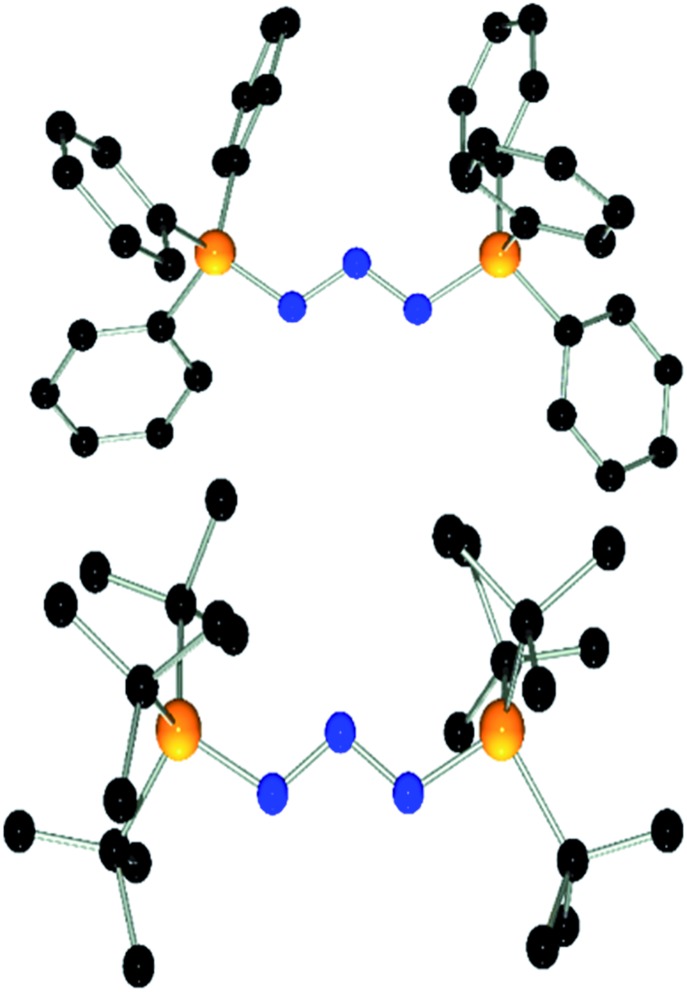
POV-ray depiction of the cations in **3** (top) and **4** (bottom). Hydrogen atoms are omitted for clarity. Selected bond distances and angles for **3**: N(1)–P(1) 1.648(3), N(3)–P(2) 1.643(3), N(1)–N(2) 1.298(4), N(2)–N(3) 1.309(4), N(2)–N(1)–P(1) 114.5(3), N(1)–N(2)–N(3) 113.2(4), N(2)–N(3)–P(2) 114.7(3), for **4**: P(1)–N(3) 1.675(2), P(2)–N(1) 1.673(2), N(1)–N(2) 1.309(2), N(3)–N(2) 1.230(2), N(2)–N(1)–P(2) 118.1(2), N(2)–N(3)–P(1) 118.9(2), N(3)–N(2)–N(1) 110.8(2).

The corresponding reaction of **1** with Ph_3_P in CH_2_Cl_2_ was monitored by ^31^P{^1^H} NMR spectroscopy revealing the complete consumption of Ph_3_P and the liberation of only 0.5 equivalents of (*p*-HC_6_F_4_)_3_P, leaving approximately 50% of **1** unreacted. Addition of another equivalent of Ph_3_P resulted in its complete consumption. Collectively, this indicates the formation of a bis-adduct [(Ph_3_P)_2_(N_3_)][B(C_6_F_5_)_4_] (**3**, Scheme 1). While the proposed intermediate [Ph_3_PN_3_]^+^ was independently synthesized by the reaction of [Ph_3_PF][B(C_6_F_5_)_4_]20*c* and Me_3_SiN_3_ (see ESI†), this species was not observed in the reaction of **1** with Ph_3_P. This indicates that reaction of [Ph_3_PN_3_]^+^ with a second equivalent of Ph_3_P is rapid, in agreement with Wiberg who described the [(Ph_3_P)_2_(N_3_)]^+^ cation in 1967.24*c* Compound **3** was isolated in high yields (97%). The *t*-Bu-substituted analog **4** was obtained from **1** with two equivalents of *t*-Bu_3_P or by reaction of **2** with one equivalent of *t*-Bu_3_P (Scheme 1). Species **4** was also obtained by reaction of **3** with two equivalents of *t*-Bu_3_P with concurrent release of Ph_3_P. All methods furnish **4** in high yields (76–99%). Compounds **3** and **4** show ^31^P{^1^H} NMR resonances at 30.6 and 56.5 ppm that are between the chemical shift ranges of phosphinimine and *N*-substituted phosphonium derivatives.^31^ X-Ray structure determination confirmed the formulations (Fig. 2). The P_2_N_3_-moiety adopts a W-shaped geometry with all five atoms almost located within one plane (largest deviation: **3**: 0.013(2) Å for N2, **4**: 0.039(1) Å for N2). The acute N–N–N angles (**3**: 113.2(4)°, **4**: 110.8(1)°) are in the typical range for phosphazides.^32^ The two P–N–N angles in each compound are **3**: 114.5(3)°/114.7(3)°; **4**: 118.1(1)°/118.9(1)° with the larger angles in **4** reflecting the increased steric congestion. Similarly, the P–N bond lengths of 1.648(3) Å and 1.643(3) Å and 1.675(2) Å and 1.673(2) Å seen in **3** and **4**, respectively, range between those of phosphinimine and *N*-substituted phosphonium derivatives.^31^ The N–N bonds in **3** and **4** were found to be 1.298(4) Å, 1.309(4) Å and 1.309(2) Å, 1.300(2) Å,^28^ which are intermediate between single and double bond distances. A similar conformation is observed for several crystallographically characterized organo-phosphazides R_3_PN_3_R,^32^ formed in the initial step of a Staudinger reaction.^33^

It is of interest to note that, similar to **1** and **2**, **4** is thermally stable and can be heated to 120 °C in C_6_D_5_Br solution over several days without decomposition. In contrast, compound **3** is not temperature stable, decomposing quantitatively with release of N_2_ within 3 h at 100 °C.24*c* The ^31^P{^1^H} NMR spectrum shows only one resonance at *δ*(^31^P) = 21.1 ppm assignable to [(Ph_3_P)_2_N][B(C_6_F_5_)_4_] (**5**) (Scheme 1).^34^ N_2_ elimination is thought to follow isomerization of the W-shaped **3** to a U-shaped isomer. It is noteworthy that compound **3** was isolated as a mixture of both isomers in a 4 : 1 ratio. The ^31^P{^1^H} NMR spectrum of the minor isomer gave two resonances at *δ*(^31^P) = 28.4 and 11.0 ppm (^4^*J*_PP_ = 5.5 Hz). At elevated temperatures, rapid conversion of the cation **3** to [(Ph_3_P)_2_N]^+^**5** was observed. Nonetheless, the ratio of the isomers of **3** is unchanged inferring the rate of N_2_-loss is comparable to the rate of isomerization of **3**. Computations at the wB97XD/def2-TZV level of theory with addition of the conductor-like polarizable continuum solvation model (CPCM)^35^ showed only a small energy difference between the W and U-shaped isomers of **3** (Δ*G*_R_^298^ = –2.6 kcal mol^–1^).

**Fig. 3:**
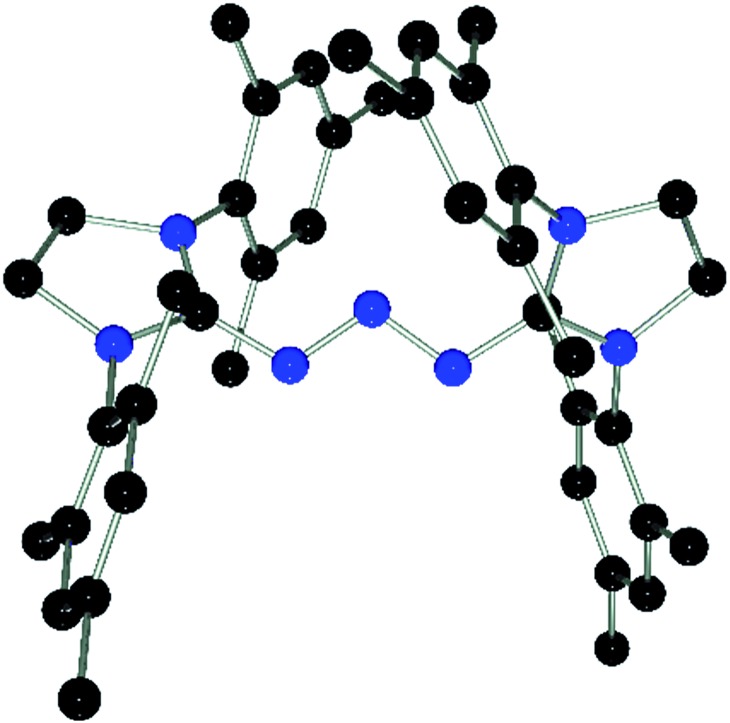
POV-ray depiction of the cation in **6**. Hydrogen atoms are omitted for clarity. Selected bond distances and angles: C(1)–N(4) 1.332(3), C(1)–N(5) 1.346(2), C(1)–N(1) 1.359(2), C(22)–N(7) 1.335(2), C(22)–N(6) 1.337(2), C(22)–N(3) 1.369(2), N(1)–N(2) 1.309(2), N(2)–N(3) 1.311(2), N(4)–C(1)–N(5) 111.1(2), N(4)–C(1)–N(1) 119.3(2), N(5)–C(1)–N(1) 129.4(2), N(7)–C(22)–N(6) 111.7(2), N(7)–C(22)–N(3) 119.9(2), N(6)–C(22)–N(3) 128.2(2), N(2)–N(1)–C(1) 113.6(2), N(1)–N(2)–N(3)110.4(2), N(2)–N(3)–C(22) 111.9(2).

Compound **1** also reacts with two equivalents of 1,3-dimesitylimidazolidin-2-ylidene (SIMes) in C_6_D_5_Br solution with liberation of (*p*-HC_6_F_4_)_3_P as evidenced by the ^31^P{^1^H} NMR spectrum. The ^13^C{^1^H} NMR spectrum shows a new resonance for the C-2 carbon atom at *δ*(^13^C) = 162.2 ppm.^36^ Collectively, the NMR data indicate the formation of [(SIMes)_2_N_3_][B(C_6_F_5_)_4_] (**6**) which was isolated in 70% yield. Interestingly, **6** was the only product obtained from the reaction using a 1 : 1 stoichiometry. This contrasts with the reactivity observed for *t*-Bu_3_P and is likely a result of the low solubility of **1** in bromobenzene. Interestingly, compound **6** was also obtained in high yields by reaction of **3** or [Ph_3_PN_3_][B(C_6_F_5_)_4_] with SIMes in 1 : 2 stoichiometry (see ESI†). Compound **6** is thermally stable even under prolonged heating to 120 °C for several days in C_6_D_5_Br solution. The nature of compound **6** was further confirmed by single crystal X-ray diffraction (Fig. 3). The structure features a W-shaped N_3_-chain terminated by imidazolidiniumyl-groups with a N–N–N angle (110.4(2)°) comparable to that seen for **3** and **4**. The planes of the imidazole-rings are skewed with respect to the N_3_-plane (38.3(7)°/41.2(5)°) and the torsion angles involving the C–N bonds (N5–C1–N1–N2: 27.6°, N6–C22–N3–N2: 35.0°) deviate from those expected for a C=N double bond. This indicates that electron delocalization across the imidazole and azide moiety may be hampered by the steric demands of the Mes substituents. The N–N (1.309(2) Å/1.311(2) Å) and C–N (1.359(2) Å/1.369(2) Å) bond distances involving the W-type fragment of **6** fall in the range between those expected for single and double bonds.^37^

**Fig. 4:**
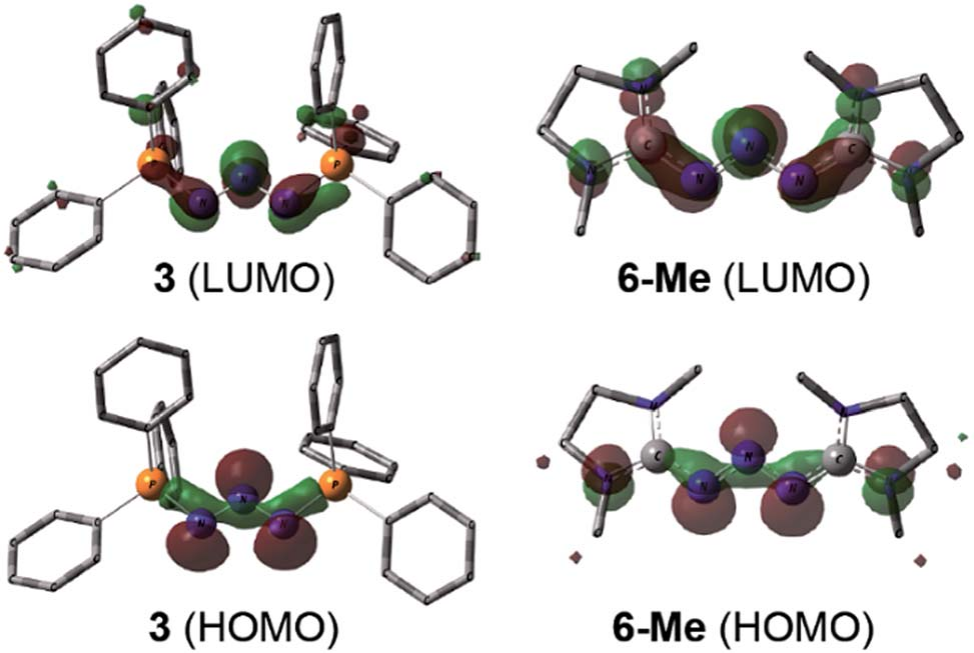
Graphical presentation of the LUMO (top) and HOMO (bottom) of the cations in **3** and **6-Me** calculated at the wB97XD/def2-TZVPP level of theory (surface iso-value = 0.05).

DFT calculations (wB97XD/def2-TZV, see ESI†) carried out on **3** and the model compound **6-Me**, (mesityl-substituents replaced by methyl groups) showed similar molecular orbitals for both compounds (Fig. 4). The HOMOs exhibit strong *n* character comprised primarily of the lone pairs of the N-atoms. The LUMOs are π-type orbitals delocalized across the N_3_-linkage and the L donors. This is in contrast to the isolated P_3_-allyl anion frontier orbitals reported for [(carbene)_2_P_3_]^+^.^15^ Interestingly these frontier orbitals are also reminiscent of the HOMO and HOMO–1 of neutral triazenes (NHC)N_3_R.^38^

## Conclusions

In summary, the EPC [(*p*-C_6_F_4_H)_3_PF][B(C_6_F_5_)_4_] is used to prepare the phosphonium ion salt **1** which serves as a precursor for the formal transfer of [N_3_]^+^ to other donors affording stable and isolable mono- and bis-adducts of the form [(L)N_3_]^+^ and [(L)_2_N_3_]^+^. The reactivity of these species containing terminal and bridging azide-fragments is the subject of continuing studies. In addition, the exploration of the reactivity of EPCs as synthetic building blocks for other unusual main group cations is ongoing.

## Supplementary Material

Supplementary information

Crystal structure data
