# Supplementary material for: Phosphine and carbene azido-cations: [(L)N3]+ and [(L)2N3]+
Source: Chem Sci. 2015 Jul 21;6(11):6367–72. doi: 10.1039/c5sc02336j (PMC6054069; doi:10.1039/c5sc02336j)
Supplement: Supplementary file 1 [file SC-006-C5SC02336J-s001.pdf]

Electronic Supplementary Material (ESI) for Chemical Science.
This journal is © The Royal Society of Chemistry 2015

Phosphine and Carbene Azido-Cations: $[(L)N_3]^+$ and $[(L)_2N_3]^+$

Daniel Winkelhaus, Michael H. Holthausen, Roman Dobrovetsky and Douglas W. Stephan

Supporting Information

This PDF file includes:

**1.**	**Materials and Methods**	S3
**2.**	**Syntheses and Spectroscopic Data**	S5
	**2.1. Preparation of [(*p*-$HC_6F_4)_3PN_3][B(C_6F_5)_4]$ (1)**	S5
	**2.2. Preparation of $[Ph_3PN_3][B(C_6F_5)_4]$**	S5
	**2.3. Preparation of [*t*-$Bu_3PN_3][B(C_6F_5)_4]_2$ (2)**	S7
	**2.4. Preparation of $[(Ph_3P)N_3(Ph_3P)][B(C_6F_5)_4]_2$ (3)**	S8
	**2.5. Preparation of [(*t*-$Bu_3P)N_3$(*t*-$Bu_3P)][B(C_6F_5)_4]_2$ (4)**	S8
	**2.6. Preparation of $[(Ph_3P)N(Ph_3P)][B(C_6F_5)_4]_2$ (3)[S8]**	S9
	**2.7. Preparation of $[(SIMes)N_3(SIMes)][B(C_6F_5)_4]_2$ (6)**	S10
	**2.8 Variable temperature NMR experiments on 3**	S11
**4.**	**Computational Details**	S13
**4.**	**Crystallographic Details**	S24
**5.**	**References**	S26

1. Materials and Methods

General Remarks

All manipulations were performed in a Glove box MB Unilab produced by MBraun or using standard Schlenk techniques[S1] under an inert atmosphere of anhydrous N_2. Dry, oxygen-free solvents (CH_2Cl_2, *n*-pentane, *n*-hexane, toluene) were prepared using an Innovative Technologies solvent purification system. Fluorobenzene (C_6H_5F) was distilled from CaH_2 and stored over molecular sieves (4 Å) prior to use. Deuterated benzene (C_6D_6) and D^8-thf were purchased from Sigma-Aldrich, distilled from sodium and stored over molecular sieves (4 Å) for at least two days prior to use. Deuterated dichloromethane (CD_2Cl_2) and bromobenzene (C_6D_5Br) were purchased from Sigma-Aldrich, distilled from CaH_2 and stored over molecular sieves (4 Å) for at least two days prior to use. Ph_3P and *t*-Bu_3P were purchased from Sigma-Aldrich and XeF_2 was purchased from Apollo Scientific and all were used without further purification. Reagents such as $[Et_3Si][B(C_6F_5)_4]*2(C_7H_8)$,[S2] 1,3-dimesityl-4,5-dihydroimidazol-3-ium-2-ylidene,[S3] $[(p\text{-}HC_6F_4)_3PF][B(C_6F_5)_4]$[S4] and $[Ph_3PF][B(C_6F_5)_4]$[S5] were prepared according to literature known procedures. All glassware was oven-dried at temperatures above 180°C prior to use. NMR spectra were measured on a Bruker AVANCE 400 (^1H (400 MHz), ^{11}B (128 MHz) ^{13}C (101 MHz), ^{19}F (377 MHz) ^{31}P (162 MHz) or a Agilent DD2 500 (1H: 500 MHz, 13C: 125 MHz, 31P: 202 MHz, 19F: 471 MHz) at ambient temperature. All ^{13}C NMR spectra were exclusively recorded with composite pulse decoupling. Assignments of the carbon atoms in the ^{13}C spectra were performed via indirect deduction from the cross-peaks in 2D correlation experiments (HMBC; HSQC). Chemical shifts were referenced to $\delta_{TMS} = 0.00$ ppm (^1H, ^{13}C) and $\delta_{H3PO4(85\%)} = 0.00$ ppm (^{31}P, externally). Chemical shifts (δ) are reported in ppm, multiplicity is reported as follows (s = singlet, d = doublet, t = triplet, quart. = quartet, m = multiplet) and coupling constants (J) are reported in Hz. Assignments of individual resonances were done using 2D techniques (HMBC, HSQC, HH-COSY) when necessary. Yields of products in solution were determined by integration of all resonances observed in the respective NMR spectra if not stated otherwise. High-resolution mass spectra (HRMS) were obtained on a micro mass 70S-250 spectrometer (EI), an ABI/Sciex QStar Mass Spectrometer (DART), or on a JOEL AccuTOF-DART (DART). Elemental analyses (C, H, N) were performed at the University of Toronto employing a Perkin Elmer 2400 Series II CHNS Analyzer.

X-ray Diffraction Studies.

Single crystals were coated with Paratone-N oil, mounted using a glass fibre pin and frozen in the cold nitrogen stream of the goniometer. Data sets were collected on a Siemens Smart System CCD diffractometer which was equipped with a rotation anode using graphite-monochromated Mo$K\alpha$ radiation ($\lambda = 0.71073$ Å) Data reduction was performed using the Bruker SMART[S6] software package. Data sets were corrected for absorption effects using SADABS routine (empirical multi-scan method). Structure solutions were found with the SHELXS-97 package using the direct method and were refined with SHELXL-97[S7] against F^2 using first isotropic and anisotropic thermal parameters for all non-hydrogen atoms. Despite several crystallization attempts involving compound [t-Bu_3PN_3][$B(C_6F_5)_4$] (**2**) only single crystals of low quality were obtained and all measured datasets suffered from low completeness (< 92%). However, these datasets were sufficient to unambiguously confirm the molecular structure of **2**. The unit cell of **6** contains 3.5 molecules CH_2Cl_2 which have been treated as a diffuse contribution to the overall scattering without specific atom positions by SQUEEZE/PLATON due to their high degree of disorder. Hydrogen atoms bonded to carbon atoms were generated with idealized geometries and isotropically refined using a riding model. Further details are given in tables S4.1 and S4.2 (pages S24 and S25).

2. Syntheses and Spectroscopic Data

2.1. Preparation of $[(p\text{-}HC_6F_4)_3PN_3][B(C_6F_5)_4]$ (1)

To a suspension of the fluorophosphonium $[(p\text{-}HC_6F_4)_3PF][B(C_6F_5)_4]$ (200 mg, 0.17 mmol, 1.0 eq.) in CH_2Cl_2 (4 mL) was added a solution of N_3SiMe_3 (38 mg, 0.34 mmol, 2.0 eq.) in CH_2Cl_2 (1 mL). The solution was stirred overnight and subsequently concentrated *in vacuo* to about half of its original volume. The addition of *n*-pentane (10 mL) initiated the formation of a white precipitate. The supernatant was removed and the residue was washed with *n*-pentane (2 x 2 mL). The residue was dried *in vacuo* yielding **1** as a white microcrystalline material (<99% yield). Single crystals of **1**, suitable for X-ray analysis, were obtained from diffusion of *n*-pentane into a CH_2Cl_2 solution.

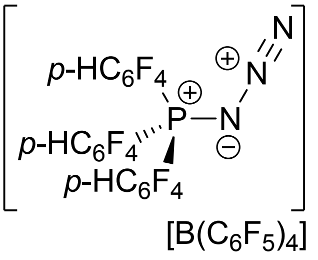

1

Yield: 203 mg; **^{1}H NMR (CD_2Cl_2, [ppm]):** δ = 7.93 ppm (m, 3H, *p*-C_6F_4*H*); **^{11}B{^{1}H} NMR (CD_2Cl_2, [ppm]):** δ = −16.7 ppm (s, $\nu_{1/2}$ = 50 Hz); **^{13}C{^{1}H} NMR (CD_2Cl_2, [ppm]):** δ = 148.5 (dm, $^{1}J_{CF}$ = 238.0 Hz, C_6F_5), 147.6 (dm, $^{1}J_{CF}$ = 260 Hz, 2X C_6F_4H), 138.6 (d, $^{1}J_{CP}$ = 242.0 Hz, C_6F_5), 136.7 (d, $^{1}J_{CP}$ = 247.0 Hz, C_6F_5), , 124.2 (br, *i*-C_6F_5), 119.4 (td, $^{2}J_{CF}$ = 22 Hz, $^{4}J_{CP}$ = 1.5 Hz, *p*-C_6F_4H), 99.7 ppm (dm, $^{1}J_{CP}$ =124.9 Hz, *i*-C_6F_4H); **^{19}F NMR (CD_2Cl_2, [ppm]):** δ = −127.9 (m, 6F, *o*-C_6F_4H), −128.9 (m, 6F, *m*-C_6F_4H), −133.3 (m, 8F, *o*-C_6F_5), −163.9 (t, $^{3}J_{FF}$ = 20.9 Hz, 4F, *p*-C_6F_5), −167.8 ppm (m, 8F, *m*-C_6F_5); **^{31}P{^{1}H} (CD_2Cl_2, [ppm]):** δ = 17.5 ppm (s); **elemental analysis** for $C_{42}H_3BF_{32}N_3P$ (1198.24): calcd 42.1, H 0.3, N 3.5; found C 41.9, H 0.3, N 3.7; **ESI MS:** m/z: 495.0 (calcd. for $[(C_6F_4H)_3PNH_3]^{2+}$: 495.0).

2.2. Preparation of $[Ph_3PN_3][B(C_6F_5)_4]$

To a suspension of the fluorophosphonium $[Ph_3PF][B(C_6F_5)_4]$ (164 mg, 0.17 mmol, 1.0 eq.) in CH_2Cl_2 (4 mL) was added a solution of N_3SiMe_3 (38 mg, 0.34 mmol, 2.0 eq.) in CH_2Cl_2 (1 mL). The colorless solution was stirred for one hour and subsequently concentrated *in vacuo* to about the half of its original volume. The addition of *n*-pentane (10 mL) initiated the formation of a white precipitate. The supernatant was removed and the residue was washed with *n*-pentane (2 x 2 mL). The residue was dried *in vacuo* and the product was obtained as a

colorless, microcrystalline material (93% yield). Single crystals, suitable for X-ray analysis, were obtained from diffusion of *n*-pentane into a CH_2Cl_2 solution of the product overnight.

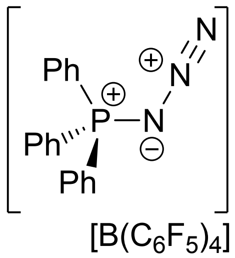

Yield: 155 mg; **(^{1}H NMR CD_2Cl_2, [ppm]):** δ = 7.98 (m, 1H, Ph), 7.80 (m, 2H, Ph), 7.73 ppm (m, 2H, Ph); **^{11}B{^{1}H} NMR (CD_2Cl_2, [ppm]):** δ = −16.7 ppm (s, $v_{1/2}$ = 50 Hz); **^{13}C{^{1}H} NMR (CD_2Cl_2, [ppm]):** δ = 148.5 (dm, $^1J_{CF}$ = 238.0 Hz, C_6F_5), 138.6 (d, $^1J_{CP}$ = 242.0 Hz, C_6F_5), 137.8 (d, $^4J_{CP}$ = 3.1 Hz, *p*-C_6H_5), 136.7 (d, $^1J_{CP}$ = 247.0 Hz, C_6F_5), 133.7 (d, $^{2/3}J_{CP}$ = 11.9 Hz, *o*/*m*-C_6H_5), 131.4 (d, $^{2/3}J_{CP}$ = 14.9 Hz, *o*/*m*-C_6H_5), 124.2 (br, *i*-C_6F_5), 117.1 ppm (d, $^1J_{CP}$ = 100.8 Hz, *i*-C_6H_5); **^{19}F NMR (CD_2Cl_2, [ppm]):** δ = −133.3 (m, 8F, *o*-C_6F_5), −163.7 (t, $^3J_{FF}$ = 20.9 Hz, 4F, *p*-C_6F_5), −167.6 ppm (m, 8F, *m*-C_6F_5); ^{31}P{^{1}H} (242.8 MHz, CD_2Cl_2, 25 °C): δ = 47.5 ppm (s); **elemental analysis** for $C_{42}H_{15}BF_{20}N_3P$ (983.35): calcd C 51.3, H 1.5, N 4.3; found C 51.2, H 1.5, N 4.3; **ESI MS:** m/z: 279.1 (calcd. for $[(C_6F_4H)_3PNH_3]^{2+}$: 279.1).

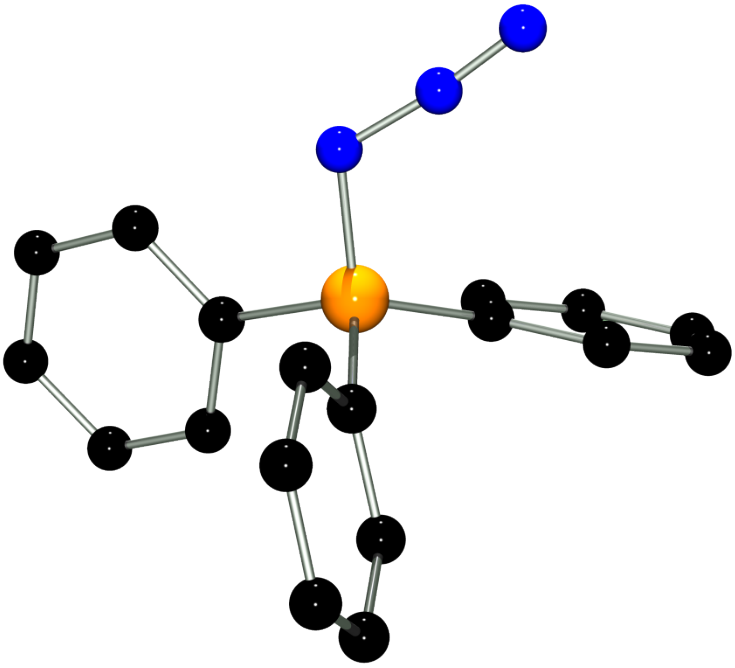

Figure 2.2.1. POV-ray depiction of the cation in $[Ph_3PN_3][B(C_6F_5)_4]$ (P: orange, N: blue, C: black).

2.3. Preparation of [*t*-Bu_3PN_3][$B(C_6F_5)_4$] (2)

A solution of *t*-Bu_3P (22 mg, 0.11 mmol, 1.0 eq.) was added to a solution of [(*p*-$C_6F_4H)_3PN_3$][$B(C_6F_5)_4$] (**1**) (129 mg, 0.11 mmol, 1.0 eq.) in CH_2Cl_2 (5 mL) in CH_2Cl_2 (2 mL). The bright yellow solution was stirred for 15 min. The addition of *n*-pentane (10 mL) initiated the formation of a yellowish precipitate. The supernatant containing P(*p*-$C_6F_4H)_3$ was removed and the residue was washed with *n*-pentane (2 x 2 mL). Removal of all volatiles *in vacuo* afforded the product as a colorless, microcrystalline solid (98% yield). Single crystals, suitable for X-ray analysis, were obtained from diffusion of *n*-pentane into a CH_2Cl_2 solution.

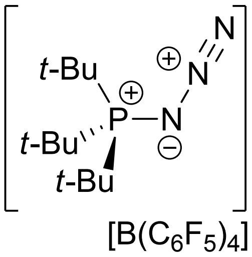

2

Yield: 97 mg; **^{1}H NMR (CD_2Cl_2, [ppm]):** δ = 1.67 ppm (d, $^3J_{HP}$ = 16.0 Hz, 9H, CH_3); **^{11}B{^{1}H} NMR (CD_2Cl_2, [ppm]):** δ = −16.7 ppm (s, $v_{1/2}$ = 50 Hz); **^{13}C{^{1}H} NMR (CD_2Cl_2, [ppm]):** δ = 148.5 (dm, $^1J_{CF}$ = 238.0 Hz, C_6F_5), 138.7 (d, $^1J_{CP}$ = 244.0 Hz, C_6F_5), 136.7 (d, $^1J_{CP}$ = 245.0 Hz, C_6F_5), 124.4 (br, *i*-C_6F_5), 44.2 (d, $^2J_{CP}$ = 26.9 Hz, *C*CH_3), 29.2 ppm (s, CH_3); **^{19}F NMR (CD_2Cl_2, [ppm]):** δ = −133.3 (m, 8F, *o*-C_6F_5), −163.7 (t, $^3J_{FF}$ = 20.9 Hz, 4F, *p*-C_6F_5), −167.6 ppm (m, 8F, *m*-C_6F_5); **^{31}P{^{1}H} (CD_2Cl_2, [ppm]):** δ = 85.9 ppm (s); **elemental analysis** for $C_{38}H_{32}BF_{20}N_3P$ (952.45): calcd C 46.8, H 3.0, N 4.6; found C 46.9, H 2.9, N 4.5; **ESI MS**: m/z: 244.2 (calcd. for [*t*$Bu_3PN_3]^+$: 244.2).

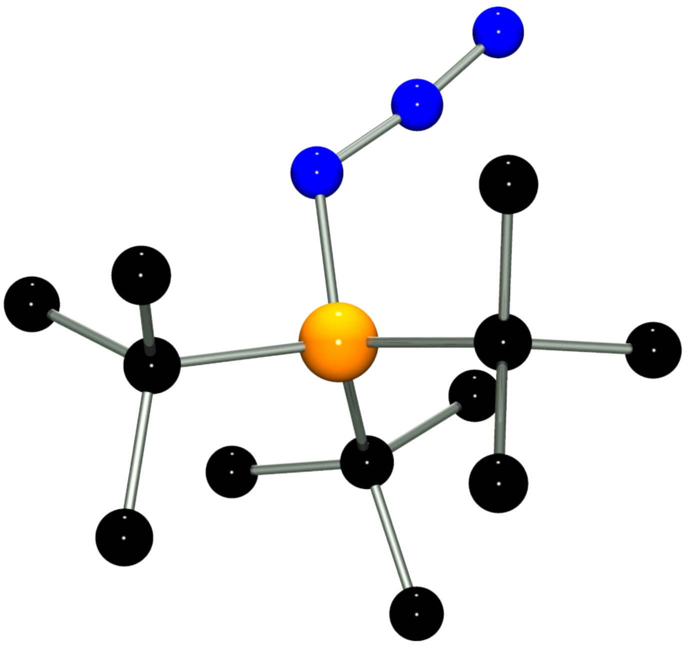

Figure 2.3.1. POV-ray depiction of the cation in [*t*-Bu_3PN_3][$B(C_6F_5)_4$] (P: orange, N: blue, C: black).

2.4. Preparation of [$(Ph_3P)N_3(Ph_3P)$][$B(C_6F_5)_4$] (3)

Route A: Ph_3P (10 mg, 0.038 mmol, 2.0 eq.) was added to a solution of [(*p*-$HC_6F_4)_3PN_3$] [$B(C_6F_5)_4$] (**1**) (23 mg, 0.019 mmol, 1.0 eq.) in CD_2Cl_2. The solution immediately turned bright yellow. The reaction mixture was investigated by means of ^{1}H, ^{13}C{^{1}H}, ^{19}F and ^{31}P NMR spectroscopy which indicated quantitative transformation of **1** to [$(Ph_3P)N_3(Ph_3P)$][$B(C_6F_5)_4$] (**4**) and (*p*-$HC_6F_4)_3P$.

Route B: A solution of [Ph_3PN_3][$B(C_6F_5)_4$] (153 mg, 0.16 mmol, 1.0 eq.) in C_6H_5F (3 mL) was added to a solution of PPh_3 (41 mg, 0.16 mmol, 1.0 eq.) in C_6H_5F (2 mL) and the bright yellow solution was stirred for one hour. The addition of *n*-pentane (10 mL) initiated the formation of a bright yellow precipitate. The supernatant was removed and the residue was washed with *n*-pentane (2 x 2 mL). The product was obtained as a bright yellow, microcrystalline solid after evaporation of the residual solvent in the glove box atmosphere (97% yield).

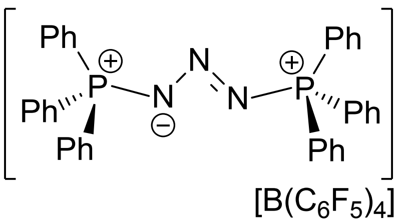

3

Yield: 188 mg; **^{1}H NMR (CD_2Cl_2, [ppm]):** δ = 7.73 (br m, 6H, Ph), 7.80 ppm (br m, 24H, Ph); **^{11}B{^{1}H} NMR (CD_2Cl_2, [ppm]):** δ = −16.7 ppm (s, $\nu_{1/2}$ = 50 Hz); **^{13}C{^{1}H} NMR (CD_2Cl_2, [ppm]):** δ = 148.5 (dm, $^{1}J_{CF}$ = 241.4 Hz, C_6F_5), 138.6 (d, $^{1}J_{CP}$ = 244.0 Hz, C_6F_5), 136.7 (d, $^{1}J_{CP}$ = 244.0 Hz, C_6F_5), 134.9 (br s, *p*-C_6H_5), 133.9 (d, $^{2/3}J_{CP}$ = 8.9 Hz, *o*/*m*-C_6H_5), 130.0 (d, $^{2/3}J_{CP}$ = 12.6 Hz, *o*/*m*-C_6H_5), 124.0(br, *i*-C_6F_5), 122.7 ppm (d, $^{1}J_{CP}$ = 96.2 Hz, *i*-C_6H_5); **^{19}F NMR (CD_2Cl_2, [ppm]):** δ = −133.1 (m, 8F, *o*-C_6F_5), −163.8 (t, $^{3}J_{FF}$ = 20.9 Hz, 4F, *p*-C_6F_5), −167.6 ppm (m, 8F, *m*-C_6F_5); **^{31}P{^{1}H} (CD_2Cl_2, [ppm]):** δ = 30.6 (s, major isomer (**3**), 78%), 28.4 (br, minor isomer (**3'**), 11%), 11.0 ppm (br, minor isomer (**3'**), 11%); **elemental analysis** for $C_{60}H_{30}BF_{20}N_3P_2$ (1245.65): calcd C 57.85, H 2.43, N 3.37; found C 57.67, H 2.41, N 3.32. **ESI MS:** m/z: 279.1 (calcd. for [$(C_6F_4H)_3PNH_3]^{2+}$: 279.1).

2.5. Preparation of [(*t*-$Bu_3P)N_3$(*t*-Bu_3P)][$B(C_6F_5)_4$] (4)

Route A: *t*-Bu_3P (17 mg, 0.077 mmol, 3 eq.) was added to a solution of [(*p*-$HC_6F_4)_3PN_3$] [$B(C_6F_5)_4$] (**1**) (21 mg, 0.026 mmol, 1 eq.) in CD_2Cl_2. The solution immediately turned bright yellow. The reaction mixture was investigated by means of ^{1}H, ^{13}C{^{1}H}, ^{19}F and ^{31}P NMR

spectroscopy which indicated quantitative transformation of **1** to [(*t*-Bu_3P)N_3(*t*-Bu_3P)][B(C_6F_5)$_4$] (**4**) and (*p*-HC_6F_4)$_3$P.

Route B: A solution of *t*Bu_3P (60 mg, 0.30 mmol, 2.0 eq.) in CH_2Cl_2 (1 mL) was added to a solution of [(Ph_3P)N_3(PPh_3)][B(C_6F_5)$_4$] (**3**) (150 mg, 0.15 mmol, 1.0 eq.) in CH_2Cl_2 (2 mL) and the bright yellow solution was stirred overnight. The addition of *n*-pentane (10 mL) initiated the formation of a bright yellow precipitate. The supernatant was removed and the residue was washed with *n*-pentane (2 x 2 mL). The residue was dried *in vacuo* and the product was obtained as a yellow, microcrystalline solid (76% yield).

Route C: A solution of *t*-Bu_3P (4.4 mg, 0.02 mmol, 1.0 eq.) in CD_2Cl_2 (0.5 mL) was added to a solution of [*t*Bu_3PN_3][B(C_6F_5)$_4$] (**2**) (20 mg, 0.02 mmol, 1.0 eq.) in CD_2Cl_2 (0.5 mL). The reaction mixture immediately turned bright yellow. The reaction mixture was investigated by means of ^{1}H, ^{13}C{^{1}H}, ^{19}F and ^{31}P NMR spectroscopy which indicated quantitative transformation of **2** to [(*t*-Bu_3P)N_3(*t*-Bu_3P)][B(C_6F_5)$_4$] (**4**).

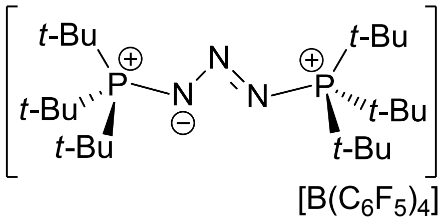

4

Yield: 130 mg (76%); **^{1}H NMR (CD_2Cl_2, [ppm]):** δ = 1.56 ppm (d, $^{3}J_{HP}$ = 1.56 Hz, 18H, CH_3); **^{11}B{^{1}H} NMR (CD_2Cl_2, [ppm]):** δ = −16.7 ppm (s, $\nu_{1/2}$ = 50 Hz); **^{13}C{^{1}H} NMR (CD_2Cl_2, [ppm]):** δ = 148.5 (dm, $^{1}J_{CF}$ = 238.0 Hz, C_6F_5), 138.6 (d, $^{1}J_{CP}$ = 244.0 Hz, C_6F_5), 136.8 (d, $^{1}J_{CP}$ = 247.0 Hz, C_6F_5), 124.4 (br, *i*-C_6F_5), 41.1 (d, $^{2}J_{CP}$ = 35.8 Hz, *C*CH_3), 30.0 ppm (s, CH_3); **^{19}F NMR (CD_2Cl_2, [ppm]):** δ = −133.1 (m, 8F, *o*-C_6F_5), −163.8 (t, $^{3}J_{FF}$ = 20.9 Hz, 4F, *p*-C_6F_5), −167.6 ppm (m, 8F, *m*-C_6F_5); **^{31}P{^{1}H} (CD_2Cl_2, [ppm]):** δ = 56.5 (s); **elemental analysis** for $C_{48}H_{54}BF_{20}N_3P_2$ (1125.71): calcd C 51.2, H 4.8, N 3.7; found C 51.0, H 5.1, N 3.6; **ESI MS**: m/z: 446.3781 (calcd. for [*t*Bu_3PN_3P*t*Bu_3]$^+$: 446.3787).

2.6. Preparation of [(Ph_3P)N_3(Ph_3P)][B(C_6F_5)$_4$] (5)[S8]

A solution of [$Ph_3PN_3PPh_3$][B(C_6F_5)$_4$] (**3**) (25 mg, 0.02 mmol, 1.0 eq.) in C_6D_5Br (1 mL) was heated to 100 °C for three hours in a J-Young NMR tube. During this time the bright yellow solution turned colorless. ^{31}P NMR spectroscopy indicated complete conversion of **3** to **5**. The addition of *n*-pentane (1 mL) initiated the formation of a white precipitate. The supernatant was removed and the residue was washed with *n*-pentane (2 x 1 mL). The residue was dried *in*

vacuo and the product was obtained as a colorless, microcrystalline solid. Compound **5** was synthesized previously.[S8]

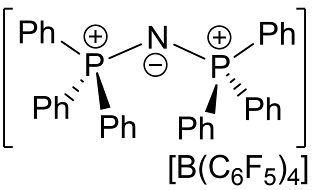

5

Yield: 23 mg (92%); **^{1}H NMR (CD_2Cl_2, [ppm]):** δ = 7.76 (m, 1H, Ph), 7.80 ppm (m, 4H, Ph); **^{11}B{^{1}H} NMR (CD_2Cl_2, [ppm]):** δ = −16.7 ppm (s, $v_{1/2}$ = 50 Hz); **^{19}F NMR (CD_2Cl_2, [ppm]):** δ = −133.1 (m, 8F, *o*-C_6F_5), −163.8 (t, $^{3}J_{FF}$ = 20.9 Hz, 4F, *p*-C_6F_5), −167.6 ppm (m, 8F, *m*-C_6F_5); **^{31}P{^{1}H} (CD_2Cl_2, [ppm]):** δ = 21.1 ppm (s).

2.7. Preparation of [(SIMes)N$_3$(SIMes)][B(C$_6$F$_5$)$_4$] (6)

Method A: SIMes (28 mg, 0.09 mmol, 2 eq.) was added to a suspension of [(*p*-$HC_6F_4)_3PN_3$] [$B(C_6F_5)_4$] (**1**) (55 mg, 0.045 mmol, 1 eq.) in C_6D_5Br (5 mL). Within two hours, **1** was completely dissolved and the color of the reaction mixture had turned orange. The reaction mixture was investigated by means of ^{1}H, ^{13}C{^{1}H}, ^{19}F and ^{31}P NMR spectroscopy which indicated quantitative transformation of **1** to [(SIMes)N$_3$(SIMes)][$B(C_6F_5)_4$] (**6**) and (*p*-$HC_6F_4)_3$P.

Method B: SIMes (30 mg, 0.1 mmol, 2 eq.) was added to a solution of [Ph_3PN_3][$B(C_6F_5)_4$] (49 mg, 0.05 mmol, 1 eq.) in C_6H_5F (5 mL). The reaction mixture turned immediately orange and investigation by means of ^{31}P{^{1}H} NMR spectroscopy revealed Ph_3P as the only P containing species present. Addition of *n*-pentane to the reaction mixture resulted in the formation of an orange precipitate. The supernatant was decanted and the precipitate was washed with *n*-pentane (3 x 3 mL). Removal of all volatiles *in vacuo* gave [(SIMes)N$_3$(SIMes)$_3$][$B(C_6F_5)_4$] (**6**) as orange, microcrystalline material (90% yield). Single crystals of **6**, suitable for X-ray single crystal structure determination were obtained by slow diffusion of *n*-pentane into a CH_2Cl_2 solution.

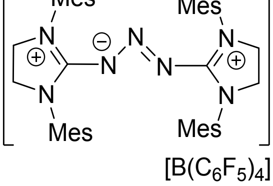

6

Yield: 60 mg (90%); **^{1}H NMR (CD_2Cl_2, [ppm]):** δ = 1.96 (24H, s, *o*-Me), 2.30 (12H, s, *p*-Me), 3.97 (8H, s, CH_2), 6.92 (8H, s, *m*-H); **^{11}B{^{1}H} (CD_2Cl_2, [ppm]):** δ = −16.7 (s); **^{13}C{^{1}H} (CD_2Cl_2, [ppm]):** δ = 17.9 (8C, s, *o*-Me), 21.1 (4C, s, *p*-Me), 48.8 (4C, s, CH_2), 130.1 (8C, s, *m*-Mes) 131.0 (4C; s, *m*-Mes), 132.1 (4C, s, *i*-Mes), 135.2 (8C, s, *o*-Mes), 136.5 (8C, d(br), C_6F_5, $^{1}J_{CF}$ = 245 Hz),138.7 (4C, d(br), C_6F_5, $^{1}J_{CF}$ = 244 Hz), 139.9 (4C, s, *p*-Mes), 148.6 (8C; d(br), C_6F_5, $^{1}J_{CF}$ = 244 Hz), 162.2 (1C, s, C-2); **^{19}F{^{1}H}**

NMR (CD_2Cl_2, [ppm]): $\delta = -167.6$ (8F, m, *m*-F), −163.8 (4F, t, *p*-F, $^3J_{FF} = 20.3$ Hz), −133.1 (8F, m, *o*-F); **elemental analysis** for $C_{66}H_{52}BF_{20}N_7$ (1339.45): calcd.: C 59.4, H 3.9, N 7.4; found: C 59.2, H 3.6, N 7.6; **ESI MS**: m/z: 654.4279 (calcd. for M^+: 654.4283).

2.8. Variable temperature NMR experiments on 3

Solutions of **3** (25 mg, 0.02 mmol, 1.0 eq.) in CD_2Cl_2 (0.7 mL) and C_6D_5Br (0.7 mL) were subjected to variable temperature NMR experiments. The CD_2Cl_2 sample was exposed to a temperature range from 25 °C to −80 °C while the C_6D_5Br sample was exposed to a temperature range from 25 °C to 70 °C.

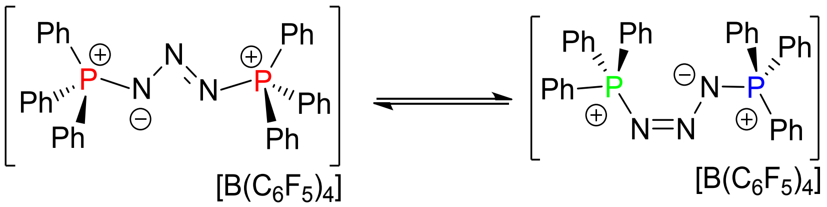

3 **3'**

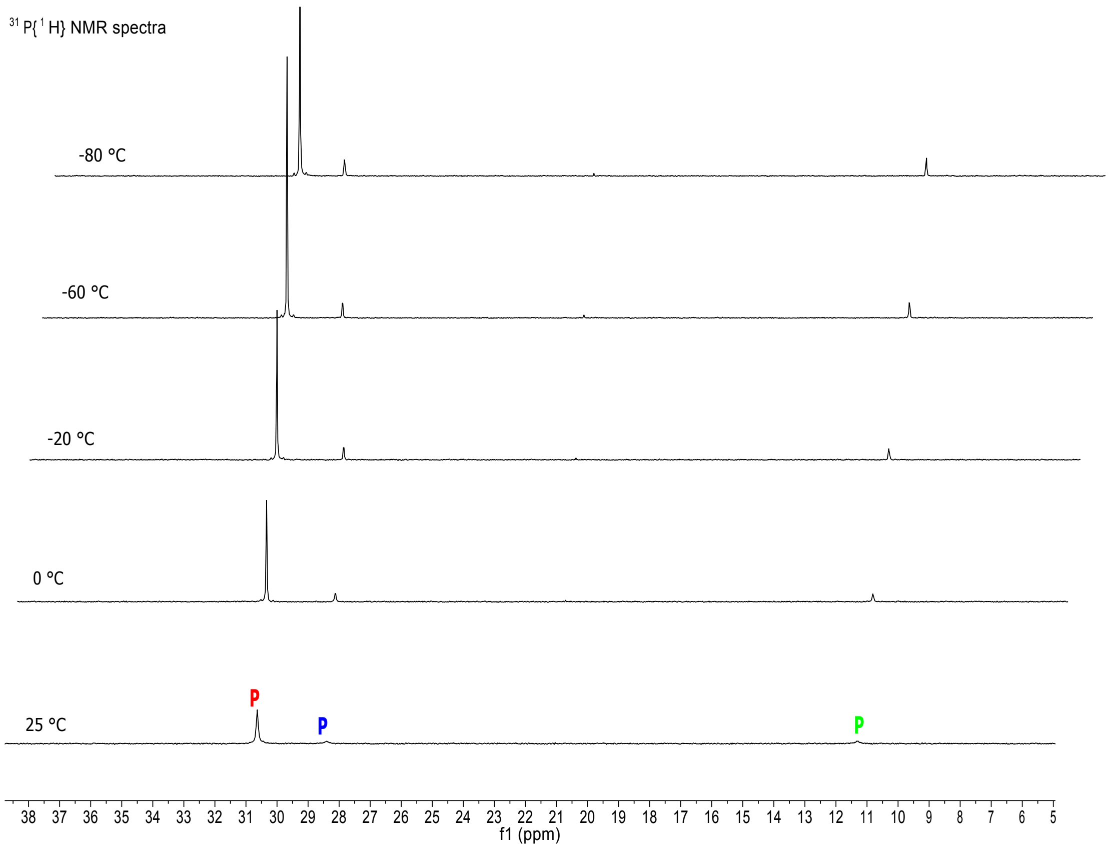

Figure S2.8.1. Variable temperature $^{31}P\{^1H\}$ NMR spectra of **3** (CD_2Cl_2, 25 °C to −80 °C).

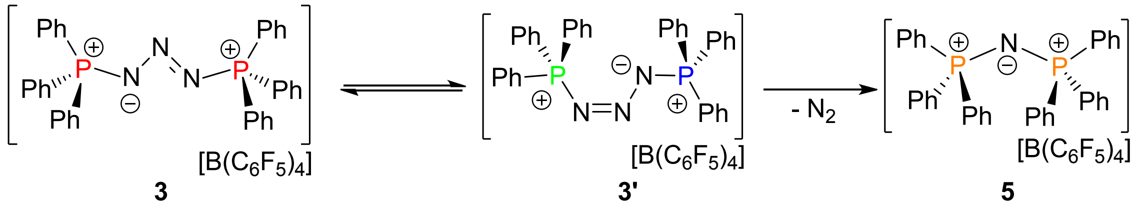

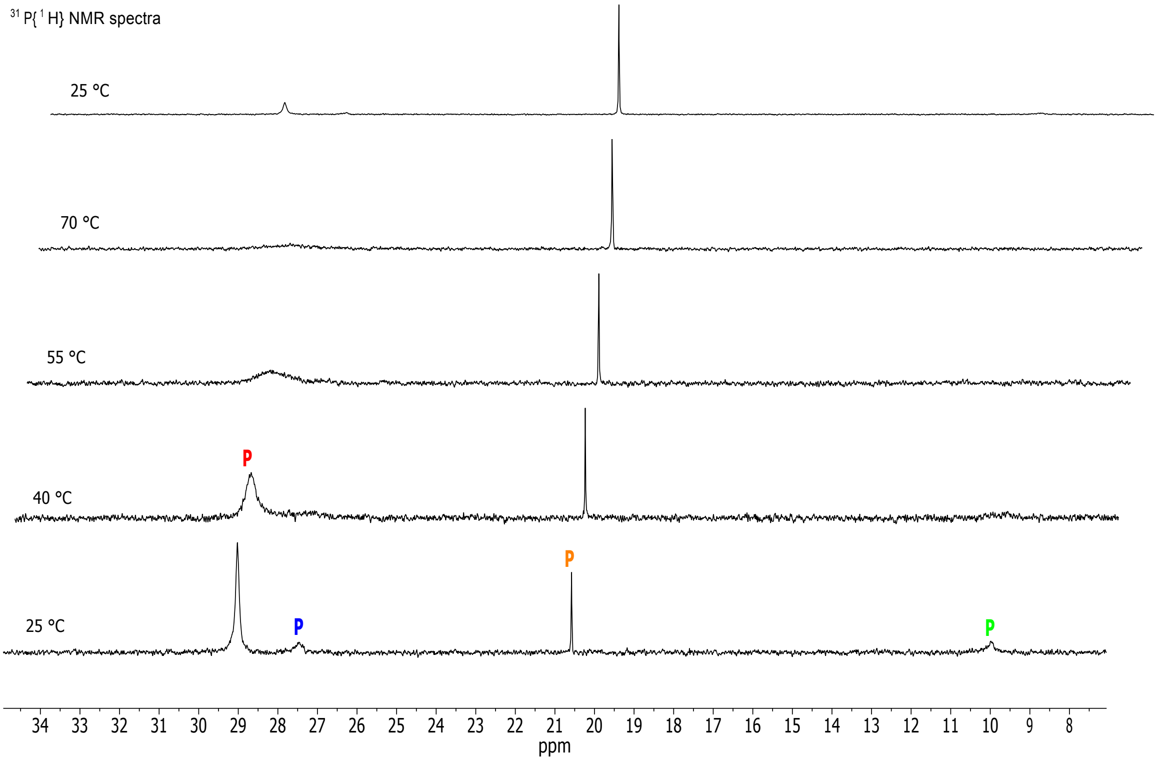

Figure S2.8.2. Variable temperature $^{31}P\{^1H\}$ NMR spectra of **3** (C_6D_5Br, 25 °C to 70 °C).

3. Computational Details

DFT calculation

DFT calculations were performed using Gaussian 09. Geometry optimization of all the molecules was carried out using the wB97XD/def2-TZV basis sets implemented in the Gaussian 09 software. The optimization of Ph_3P-N_3-PPh_3 isomers was used in conjunction with the conductor-like polarizable continuum solvation model (CPCM) implemented in the Gaussian 09 software. Thermal energy corrections were extracted from the results of frequency analysis performed at the same level of theory. Frequency analysis of all calculated molecules contained no imaginary frequency showing that these are energy minima. NBO calculations for $(HC_6F_4)_3P$-N_3 were done using NBO implemented in Gaussian 09 software.

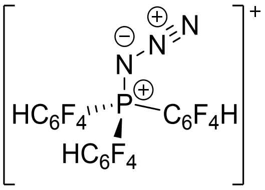

C	-0.68642800	1.58172700	0.05043200
C	-0.14114200	2.72680800	0.61142900
C	-0.60877100	3.98016200	0.28236300
C	-1.63033300	4.12075500	-0.63758800
H	-1.99797900	5.10017400	-0.90507000
C	-2.16433000	2.98897100	-1.21781800
C	-1.70011400	1.72865900	-0.88426200
C	1.67881400	-0.27481500	-0.06689500
C	2.43621000	-1.32782500	0.43752200
C	3.72516400	-1.55421400	0.00319500
C	4.28812900	-0.74798000	-0.96678100
H	5.29526600	-0.92592900	-1.31337400
C	3.53892500	0.28542300	-1.48901800
C	2.24722500	0.52304600	-1.05341000
C	-1.15779700	-1.38670100	0.15786200
C	-0.82731300	-2.45066400	-0.66962900
C	-1.74651300	-3.45429400	-0.91647300
C	-3.00655600	-3.41701800	-0.35692600
H	-3.71881000	-4.20382500	-0.55557500
C	-3.34413300	-2.35198200	0.45490600
C	-2.43753500	-1.34181300	0.69897300
N	-0.03062100	-0.12186200	2.32767100
N	0.96166300	0.28934500	2.99949100

N	1.75779300	0.55633700	3.75481900
P	-0.01553400	-0.03358000	0.58421400
F	0.40208600	-2.51554700	-1.26557400
F	-1.39028600	-4.48490900	-1.73035600
F	-2.80390900	-0.26242600	1.45400600
F	-4.58557100	-2.27809600	1.00454900
F	1.87564400	-2.15756300	1.37193000
F	4.43212000	-2.58850200	0.52935800
F	4.06352400	1.08902300	-2.45238900
F	1.53928400	1.54204600	-1.62609900
F	0.91025000	2.58703800	1.48699100
F	-0.04610000	5.07618900	0.85711100
F	-3.15820100	3.09976900	-2.13934200
F	-2.23310100	0.63709600	-1.51133200

Sum of electronic and zero-point Energies= -2390.379974 [Ha]

Sum of electronic and thermal Energies= -2390.350283 [Ha]

Sum of electronic and thermal Enthalpies= -2390.349339 [Ha]

Sum of electronic and thermal Free Energies= -2390.442233 [Ha]

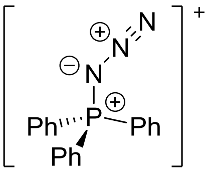

C	-0.75209900	-1.46478300	-0.28794300
C	-2.14424100	-1.57263900	-0.22080700
H	-2.74817500	-0.77743500	0.19786300
C	-2.75989800	-2.71295200	-0.71802400
H	-3.83469700	-2.80807100	-0.66806700
C	-1.99219500	-3.72876600	-1.28323500
H	-2.47579900	-4.61329000	-1.67231600
C	-0.60770300	-3.61090400	-1.35280700
H	-0.01723000	-4.39895600	-1.79668100
C	0.02194700	-2.47782100	-0.85141700
H	1.09791700	-2.38788600	-0.90857000
C	-0.76857300	1.53781700	-0.13116800
C	-0.62730300	2.68751600	0.64726700
H	-0.08433600	2.65973000	1.58283500
C	-1.20309300	3.87322000	0.21044100
H	-1.10676800	4.76776500	0.80817600
C	-1.89810100	3.90879800	-0.99607400
H	-2.34240200	4.83454400	-1.33256500
C	-2.02360700	2.76025200	-1.77179800

H	-2.56114000	2.79274000	-2.70821700
C	-1.45939800	1.56456200	-1.34265500
H	-1.56279500	0.67299300	-1.94615900
C	1.84556200	0.04451100	0.10272800
C	2.32826900	0.81652100	-0.95205100
H	1.66355000	1.42733300	-1.54834300
C	3.69056100	0.80405000	-1.22637600
H	4.07912000	1.40054800	-2.03872400
C	4.55047900	0.03337100	-0.44983200
H	5.60974100	0.03067000	-0.66386200
C	4.05692800	-0.72999900	0.60526400
H	4.72981500	-1.31944000	1.21064000
C	2.69724500	-0.73056700	0.88857300
H	2.31243400	-1.30912600	1.71668300
N	0.01085800	-0.06232400	2.18645700
N	-1.08812600	-0.35304700	2.73745500
N	-1.97254400	-0.59839900	3.40261700
P	0.05738400	0.00740900	0.41740600

Sum of electronic and zero-point Energies= -1199.636575 [Ha]

Sum of electronic and thermal Energies= -1199.617766 [Ha]

Sum of electronic and thermal Enthalpies= -1199.616822 [Ha]

Sum of electronic and thermal Free Energies= -1199.686950 [Ha]

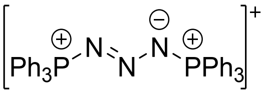

N	-1.10835300	-0.00130400	1.58166300
N	0.00022500	-0.00030500	0.87068300
N	1.10880500	0.00108300	1.58168400
P	2.46492800	0.01342000	0.45142900
P	-2.46460100	-0.01358900	0.45167200
C	3.93330700	0.11363800	1.52490500
C	3.87521800	-0.45006600	2.79794700
C	5.09833300	0.71853400	1.05666100
C	5.00615400	-0.41086300	3.60564700
H	2.95582100	-0.89114900	3.15625600
C	6.22359600	0.74908400	1.87173900
H	5.13144800	1.16917600	0.07308200
C	6.17639700	0.18357500	3.14296700
H	4.97056100	-0.84083200	4.59619700

H	7.13081300	1.21733300	1.51800400
H	7.05224900	0.21113300	3.77582900
C	2.38328500	1.48329400	-0.64070700
C	2.57307000	1.37481300	-2.01540300
C	2.13126700	2.72117900	-0.04805400
C	2.51744600	2.52082000	-2.80430500
H	2.76614400	0.41367100	-2.47165800
C	2.07572500	3.85848000	-0.84272900
H	1.97368700	2.79554900	1.01991600
C	2.27350200	3.75843900	-2.21842400
H	2.67235900	2.44429700	-3.87110100
H	1.88408600	4.82055500	-0.38987700
H	2.23998300	4.64778600	-2.83241300
C	2.53864300	-1.49652200	-0.58507900
C	1.41601200	-1.87144800	-1.32479700
C	3.70835000	-2.25035800	-0.63548300
C	1.47443500	-3.00537600	-2.12412600
H	0.50352300	-1.29479300	-1.26537100
C	3.75718500	-3.38433400	-1.43988100
H	4.57441600	-1.96529200	-0.05399300
C	2.64346800	-3.76000000	-2.18288600
H	0.60503800	-3.29884800	-2.69435900
H	4.66256900	-3.97263300	-1.48130900
H	2.68542600	-4.64199400	-2.80648100
C	-2.53904000	1.49716400	-0.58365300
C	-3.71003600	2.24891300	-0.63552900
C	-1.41591000	1.87444300	-1.32138600
C	-3.75966000	3.38312300	-1.43954800
H	-4.57649800	1.96206700	-0.05551000
C	-1.47513400	3.00861200	-2.12034000
H	-0.50248900	1.29936600	-1.26074700
C	-2.64544400	3.76111300	-2.18063900
H	-4.66602400	3.96982700	-1.48214100
H	-0.60537700	3.30390300	-2.68907900
H	-2.68801400	4.64328100	-2.80394900

C	-3.93271900	-0.11516500	1.52538400
C	-5.09754400	-0.72045600	1.05711000
C	-3.87472200	0.44819000	2.79857200
C	-6.22267100	-0.75176300	1.87232900
H	-5.13054900	-1.17082300	0.07340500
C	-5.00553800	0.40823100	3.60642500
H	-2.95547100	0.88952300	3.15695400
C	-6.17556600	-0.18658900	3.14372200
H	-7.12972800	-1.22031900	1.51858900
H	-4.96998300	0.83792200	4.59709500
H	-7.05134100	-0.21471700	3.77666400
C	-2.38305400	-1.48251200	-0.64174400
C	-2.13287100	-2.72121200	-0.05002700
C	-2.57091000	-1.37247400	-2.01657300
C	-2.07729800	-3.85775400	-0.84577200
H	-1.97677900	-2.79683500	1.01807500
C	-2.51528400	-2.51773700	-2.80656100
H	-2.76272400	-0.41074000	-2.47210800
C	-2.27319500	-3.75615900	-2.22162000
H	-1.88707600	-4.82045000	-0.39364800
H	-2.66884300	-2.43998500	-3.87346400
H	-2.23965900	-4.64492500	-2.83644600

Sum of electronic and zero-point Energies= -2235.482909 [Ha]

Sum of electronic and thermal Energies= -2235.448513 [Ha]

Sum of electronic and thermal Enthalpies= -2235.447569 [Ha]

Sum of electronic and thermal Free Energies= -2235.554260 [Ha]

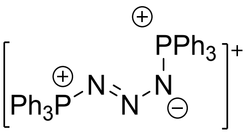

N	-1.29975600	-0.05813800	1.89552300
N	-0.02101100	-0.04222500	1.72414200
N	0.42772600	0.03491000	0.48182500
P	2.16115800	0.04726400	0.31112700
P	-2.30116900	-0.03113500	0.36354200
C	3.07060700	-0.05684000	1.89462900

C	3.08842400	-1.27191600	2.57997600
C	3.70182800	1.06962000	2.41471000
C	3.74632500	-1.35435600	3.80003800
H	2.59614500	-2.14438500	2.17197700
C	4.35927700	0.97636400	3.63717300
H	3.68928900	2.00912900	1.88008100
C	4.38196900	-0.23177900	4.32659500
H	3.76451600	-2.29158000	4.33703400
H	4.85348200	1.84551700	4.04638600
H	4.89350000	-0.30014200	5.27617800
C	2.54101400	1.61565100	-0.55317800
C	3.55494200	1.67125600	-1.50592400
C	1.80501900	2.75103500	-0.22059800
C	3.83622300	2.88222000	-2.12892800
H	4.11711000	0.78598100	-1.76991100
C	2.09442600	3.95748000	-0.84672800
H	1.00448600	2.69486300	0.50414200
C	3.10771500	4.02211500	-1.79926300
H	4.61810400	2.93297600	-2.87273100
H	1.52462200	4.84094300	-0.59762700
H	3.32803900	4.96082500	-2.28764300
C	2.60012600	-1.37241200	-0.75563700
C	1.63681700	-1.88693200	-1.62073300
C	3.89203600	-1.89486700	-0.72611200
C	1.97506200	-2.93725500	-2.46555500
H	0.63275700	-1.48630100	-1.62137900
C	4.21982200	-2.94630600	-1.57481400
H	4.63445600	-1.50034600	-0.04543800
C	3.26341200	-3.46448000	-2.44395300
H	1.23068900	-3.34305500	-3.13510600
H	5.21754400	-3.36026000	-1.55424800
H	3.52221700	-4.28166400	-3.10234600
C	-2.24184500	1.49517800	-0.67029700
C	-3.26540600	2.43400300	-0.54916000
C	-1.19708000	1.70203200	-1.57046400

C	-3.23667000	3.58563600	-1.32911800
H	-4.08174900	2.27717500	0.14097400
C	-1.18116000	2.84913900	-2.35286200
H	-0.39463800	0.98462600	-1.63823600
C	-2.19873700	3.79220200	-2.23190700
H	-4.02715400	4.31582300	-1.23212800
H	-0.36985600	3.00852300	-3.04870600
H	-2.18099300	4.68705000	-2.83798200
C	-3.98033900	-0.15265900	1.09047300
C	-4.98734000	-0.82452000	0.40072600
C	-4.24185600	0.45734000	2.31645400
C	-6.26692400	-0.88311600	0.94216900
H	-4.78311000	-1.30336200	-0.54732200
C	-5.52412100	0.39375700	2.85077300
H	-3.45299600	0.96590400	2.85097100
C	-6.53467100	-0.27493000	2.16539400
H	-7.05015700	-1.40519900	0.41159200
H	-5.73103800	0.86332000	3.80170100
H	-7.52935300	-0.32448000	2.58557800
C	-2.01438500	-1.52592200	-0.66626000
C	-1.66292000	-2.70902600	-0.01775300
C	-2.16675000	-1.48889400	-2.04938100
C	-1.45136900	-3.86050600	-0.76528900
H	-1.54271300	-2.73229300	1.05720900
C	-1.95705400	-2.64934400	-2.79019600
H	-2.44694800	-0.57426400	-2.55235400
C	-1.59957100	-3.83147700	-2.15025000
H	-1.17333700	-4.77839300	-0.26770900
H	-2.07724100	-2.62562800	-3.86379500
H	-1.43530900	-4.73002300	-2.72812000

Sum of electronic and zero-point Energies= -2235.487903 [Ha]

Sum of electronic and thermal Energies= -2235.452938 [Ha]

Sum of electronic and thermal Enthalpies= -2235.451994 [Ha]

Sum of electronic and thermal Free Energies= -2235.559154 [Ha]

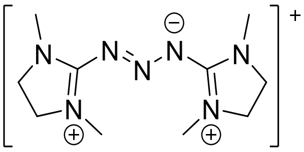

C	2.25508100	0.26059400	-0.06841800
C	4.55133400	-0.03461900	0.10380000
H	5.11036800	-0.26332200	1.00946600
H	5.20964300	0.49326400	-0.58811500
C	3.91512600	-1.29282500	-0.53075200
H	4.33880100	-1.52427000	-1.50648400
H	4.00935900	-2.17069300	0.10937900
C	-2.25510800	0.26072100	0.06858200
C	-3.91514200	-1.29290400	0.53014900
H	-4.33909800	-1.52468900	1.50567500
H	-4.00908000	-2.17058600	-0.11028500
C	-4.55127900	-0.03457900	-0.10423700
H	-5.11016700	-0.26310900	-1.01003300
H	-5.20968900	0.49316900	0.58768100
N	1.09191300	0.97790200	0.02895000
N	-0.00003400	0.24630500	0.00013600
N	-1.09188200	0.97806500	-0.02832200
N	3.38180100	0.79490300	0.41878000
N	2.49480700	-0.91653100	-0.67161300
N	-2.49490500	-0.91649500	0.67155400
N	-3.38169600	0.79501300	-0.41888700
C	-3.50574700	2.08561400	-1.07412300
H	-3.91493600	1.96107500	-2.07717600
H	-4.16680900	2.73398400	-0.49668300
H	-2.52420700	2.54564200	-1.13801600
C	-1.56097600	-1.78505000	1.36929000
H	-1.06181500	-2.47228500	0.68540700
H	-0.80523800	-1.19978700	1.88533100
H	-2.11933800	-2.36112000	2.10566000
C	1.56067100	-1.78538900	-1.36869700
H	2.11885100	-2.36181300	-2.10492400

H	1.06168000	-2.47230400	-0.68436100
H	0.80478800	-1.20037800	-1.88481400
C	3.50602700	2.08553100	1.07390000
H	2.52458800	2.54581200	1.13755300
H	3.91497300	1.96103000	2.07706200
H	4.16738000	2.73366700	0.49652500

Sum of electronic and zero-point Energies= -775.505201 [Ha]

Sum of electronic and thermal Energies= -775.486726 [Ha]

Sum of electronic and thermal Enthalpies= -775.485781 [Ha]

Sum of electronic and thermal Free Energies= -775.553137 [Ha]

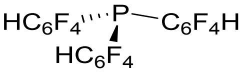

C	-1.40652600	-0.89554400	-0.31542600
C	-2.36188500	-1.57734600	-1.05397100
C	-3.44995300	-2.17560100	-0.44243700
C	-3.61775900	-2.11557800	0.92343400
H	-4.46576900	-2.58312500	1.39825800
C	-2.66953100	-1.44429800	1.66789700
C	-1.58252600	-0.85019700	1.05941900
C	1.54233900	-0.83462000	-0.58672600
C	2.73334700	-0.22175200	-0.95364300
C	3.96356700	-0.74607300	-0.62201300
C	4.05654600	-1.92961800	0.08126300
H	5.01507000	-2.34755900	0.34421000
C	2.88544200	-2.56188100	0.43429700
C	1.64969800	-2.03164400	0.10256700
C	-0.10046400	1.61547800	-0.36906200
C	0.73637200	2.12111300	0.61456700
C	0.56333500	3.39923000	1.11678300
C	-0.45799400	4.21282400	0.67733900
H	-0.59006600	5.20479000	1.07891900
C	-1.30818200	3.71605200	-0.28755900
C	-1.12958600	2.44548800	-0.79661600

P	-0.02309900	-0.04658100	-1.25188100
F	1.75736400	1.37024700	1.13725800
F	1.42103700	3.85687000	2.08412600
F	-2.01161000	1.98227900	-1.74602800
F	-2.34515900	4.48808900	-0.74494700
F	2.67752300	0.96425300	-1.65065700
F	5.10464200	-0.08458400	-0.99505900
F	2.93724800	-3.74507200	1.12641700
F	0.53312100	-2.74396000	0.47145400
F	-2.24148300	-1.67664200	-2.41526400
F	-4.37199000	-2.83968200	-1.20966400
F	-2.80243600	-1.37593200	3.03147000
F	-0.64605400	-0.23270800	1.85046600

Sum of electronic and zero-point Energies= -2226.587335 [Ha]

Sum of electronic and thermal Energies= -2226.560795 [Ha]

Sum of electronic and thermal Enthalpies= -2226.559851 [Ha]

Sum of electronic and thermal Free Energies= -2226.645794 [Ha]

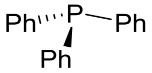

C	-0.60044100	1.55801200	-0.45618300
C	-1.73984300	2.16393100	-0.99221000
H	-2.21184700	1.73620700	-1.86782300
C	-2.27100500	3.30976300	-0.41138600
H	-3.15651100	3.76633000	-0.83246200
C	-1.65880500	3.87212200	0.70558000
H	-2.06746300	4.76627600	1.15636800
C	-0.51726200	3.28196400	1.23619400
H	-0.03457800	3.71615400	2.10146200
C	0.00898400	2.12795900	0.66013500
H	0.89373200	1.67509900	1.08543800
C	-1.04539100	-1.30318700	-0.44735100
C	-1.03225400	-2.58774900	-0.99532300
H	-0.44569700	-2.78560500	-1.88374000
C	-1.77033800	-3.61243800	-0.41317500
H	-1.75051000	-4.60317300	-0.84665400

C	-2.53984200	-3.35978200	0.71891400
H	-3.12009800	-4.15367700	1.16924500
C	-2.56361900	-2.08106500	1.26544400
H	-3.16054400	-1.87784600	2.14441200
C	-1.81768800	-1.05702500	0.68724000
H	-1.83958600	-0.06818900	1.12394200
C	1.65679600	-0.25940800	-0.44820500
C	1.82192100	-1.00450000	0.71912400
H	0.97079000	-1.48938200	1.17688900
C	3.08037100	-1.12763100	1.30228900
H	3.19707600	-1.70881300	2.20715900
C	4.18301400	-0.50505600	0.72657700
H	5.15950600	-0.60095400	1.18165000
C	4.02584900	0.23779100	-0.43991900
H	4.88019300	0.71888300	-0.89652000
C	2.77019500	0.35406600	-1.02635600
H	2.65407700	0.92076100	-1.94161800
P	0.00584100	-0.00391200	-1.29507200

Sum of electronic and zero-point Energies= -1035.777030 [Ha]

Sum of electronic and thermal Energies= -1035.761458 [Ha]

Sum of electronic and thermal Enthalpies= -1035.760513 [Ha]

Sum of electronic and thermal Free Energies= -1035.823262 [Ha]

4. Crystallographic Details

Table S4.1. Crystallographic data and details of the structure refinements of compounds [(p-$HC_6F_4)_3PN_3$][$B(C_6F_5)_4$] (**1**), [Ph_3PN_3][$B(C_6F_5)_4$], and [t-Bu_3PN_3][$B(C_6F_5)_4$] (**2**).

	[(p-$HC_6F_4)_3PN_3$] [$B(C_6F_5)_4$] (**1**)	[Ph_3PN_3][$B(C_6F_5)_4$]	[t-Bu_3PN_3][$B(C_6F_5)_4$] (**2**)
formula	$C_{42}H_3BF_{32}N_3P$	$C_{42}H_{15}BF_{20}N_3P$	$C_{36}H_{27}BF_{20}N_3P$
M_r [g mol^{-1}]	1199.25	983.35	923.39
color, habit	colorless, block	colorless, block	colorless, block
crystal system	triclinic	monoclinic	triclinic
Space group	P-1	$P2_1$/n	P-1
a [Å]	10.701(1)	12.2260(4)	10.362(1)
b [Å]	11.209(1)	23.244(1)	12.929(2)
c [Å]	17.810(1)	14.1160(4)	14.089(2)
α [°]	90.007(2)	90	94.96(1)
β [°]	104.509(2)	107.089(1)	96.26(1)
γ [°]	98.160(2)	90	100.77(1)
V [Å^3]	2045.9(2)	3834.4(2)	1832.2(4)
Z	2	4	2
T [K]	149(2)	149(2)	149(2)
Crystal size [mm]	0.40x0.30x0.20	0.40x0.30x0.20	0.30x0.30x0.30
ρ_c [g cm^{-3}]	1.947	1.703	1.674
F(000)	1168	1952	928
θ_{min} [°]	1.84	1.75	1.46
θ_{max} [°]	27.54	27.50	27.52
Index range	$-13 \leq h \leq 13$<br>$-14 \leq k \leq 14$<br>$-23 \leq l \leq 23$	$-15 \leq h \leq 15$<br>$-30 \leq k \leq 21$<br>$-18 \leq l \leq 14$	$-12 \leq h \leq 13$<br>$-14 \leq k \leq 16$<br>$-17 \leq l \leq 18$
μ [mm^{-1}]	0.255	0.208	0.211
absorption correction	SADABS	SADABS	SADABS
reflections collected	34643	34934	17985
reflections unique	9340	8786	7773
R_{int}	0.0311	0.0328	0.0682
reflection obs. [F>3σ(F)]	7059	6427	3963
residual density [e Å^{-3}]	0.409, –0.387	0.324, –0.366	0.591, –0.619
parameters	712	604	559
GOOF	1.033	1.009	1.084
R_1 [I>2σ(I)]	0.0366	0.0367	0.1117
wR_2 (all data)	0.0844	0.0876	0.3683
CCDC	1403531	1403535	-[a]

[a] several attempts for the crystallization of **2** resulted in all cases in the formation of single crystals of low quality and all measured datasets (crystallographic details see table) suffered from low completeness (< 92%) and, therefore, were not deposited in the CCDC database.

Table S4.2. Crystallographic data and details of the structure refinements of compounds $[(Ph_3P)N_3(Ph_3P)][B(C_6F_5)_4]$ (**3**), $[(t\text{-}Bu_3P)N_3(t\text{-}Bu_3P)][B(C_6F_5)_4]$ (**4**), and $[(SIMes)N_3(SIMes)][B(C_6F_5)_4]*(CH_2Cl_2)$ (**6**).

	$[(Ph_3P)N_3(Ph_3P)][B(C_6F_5)_4]$ (**3**)	$[(t\text{-}Bu_3P)N_3(t\text{-}Bu_3P)][B(C_6F_5)_4]$ (**4**)	$[(SIMes)N_3(SIMes)][B(C_6F_5)_4]*(CH_2Cl_2)$ (**6**)
formula	$C_{60}H_{30}BF_{20}N_3P_2$	$C_{48}H_{54}BF_{20}N_3P_2$	$C_{67.75}H_{55.5}BCl_{3.5}F_{20}N_7$
M_r [g mol^{-1}]	1245.62	1125.69	1482.58
color, habit	yellow, block	yellow, block	orange, block
crystal system	monoclinic	triclinic	triclinic
Space group	$P2_1/n$	P-1	P-1
a [Å]	12.841(1)	12.773(1)	11.704(1)
b [Å]	18.514(1)	14.922(1)	16.501(1)
c [Å]	22.501(1)	15.104(1)	18.334(1)
α [°]	90	107.504(3)	86.770(2)
β [°]	90.649(3)	95.932(3)	76.082(2)
γ [°]	90	111.054(2)	83.169(2)
V [Å^3]	5348(1)	2489.0(2)	3411.0(2)
Z	4	2	2
T [K]	149(2)	149(2)	149(2)
Crystal size [mm]	0.20x0.10x0.10	0.12x0.07x0.02	0.10x0.10x0.10
ρ_c [g cm^{-3}]	1.547	1.502	1.443
F(000)	2504	1156	1511
θ_{min} [°]	1.42	1.46	1.67
θ_{max} [°]	25.00	27.47	27.52
Index range	$-15 \le h \le 15$<br>$-22 \le k \le 21$<br>$-25 \le l \le 26$	$-16 \le h \le 15$<br>$-19 \le k \le 19$<br>$-19 \le l \le 18$	$-15 \le h \le 15$<br>$-21 \le k \le 21$<br>$-23 \le l \le 23$
μ [mm^{-1}]	0.196	0.201	0.256
absorption correction	SADABS	SADABS	SADABS
reflections collected	40340	41878	72578
reflections unique	9424	11238	15624
R_{int}	0.1605	0.0277	0.0337
reflection obs. [F>3σ(F)]	7059	8889	11600
residual density [e Å^{-3}]	0.428, –0.439	0.371, –0.329	0.488, –0.389
parameters	775	696	847
GOOF	0.928	1.019	1.055
R_1 [I>2σ(I)]	0.0676	0.0357	0.0534
wR_2 (all data)	0.1120	0.0886	0.1609
CCDC	1403532	1403533	1403534

5. References

[S1] D. F. Shriver, M. A. Drezdzon, *The manipulation of air sensitive compounds*, **1986**, Wiley VCH, New York, USA.

[S2] J. B. Lambert, S. Zhang, S. M. Ciro, *Organometallics* **1994**, 2430.

[S3] A. J. Arduengo, R. Krafczyk, R. Schmutzler, H. A. Craig, J. R. Goerlich, W. J. Marshall, M. Unverzagt, *Tetrahedron*,**1999**, *55*, 14523.

[S4] C. B. Caputo, D. Winkelhaus, R. Dobrovetsky, L. J. Hounjet, D. W. Stephan, *Dalton Trans.* **2015**, *44*, 12256.

[S5] M. H. Holthausen, R. R. Hiranandani, D. W. Stephan, *Chem. Sci.* **2015**, *6*, 2016.

[S6] a) *SAINT 7.23A*, Bruker AXS, Inc: Madison, Wisconsin, **2006**; b) G. M. Sheldrick, *SADABS*, Bruker AXS, Inc.: Madison, Wisconsin, **2004**.

[S7] G. M. Sheldrick, *SHELXL-97, Program for crystal structure determination,* University of Göttingen, Germany, **1997**.

[S8] P. Vanýsek, C. Zheng, *Acta Cryst.* **2013**, *E69*, o87.
